# Editorial: *Aspergillus*-derived mycotoxins in the feed and food chain, volume II

**DOI:** 10.3389/fmicb.2023.1211341

**Published:** 2023-05-10

**Authors:** István Pócsi, Federica Giacometti, Árpád Ambrus, Antonio F. Logrieco

**Affiliations:** ^1^Department of Molecular Biotechnology and Microbiology, Institute of Biotechnology, Faculty of Science and Technology, University of Debrecen, Debrecen, Hungary; ^2^Department of Veterinary Medical Sciences, University of Bologna, Bologna, Italy; ^3^Doctoral School of Nutrition and Food Sciences, University of Debrecen, Debrecen, Hungary; ^4^Institute of Sciences of Food Production, Italian National Research Council, Bari, Italy

**Keywords:** mycotoxins, risk assessment, climate change, lactic acid bacteria, biocontrol, bZIP-type transcription factors, cytochrome P450, nephropathy

Owing to the outstanding success of the previous Frontiers in Microbiology Research Topic titled “*Aspergillus-derived mycotoxins in the feed and food chain*”, we decided to re-open this chapter to collect additional and comprehensive information on *Aspergillus* mycotoxins. The major topics discussed earlier covered almost every aspect of mycotoxin research in this exceptionally important field, concentrated in 20 significant research and review papers (Pócsi et al., 2020). Research Topic “*Aspergillus-derived mycotoxins in the feed and food chain, volume II*” attracted altogether eight new papers on (i) the assessment of aflatoxin risks and the newly emerging mycotoxin removal techniques, which will hopefully be suitable for the treatment of the unfavorable trends observed in Europe recently due to the climate change, (ii) the rise of non-aflatoxigenic *Aspergillus flavus*-based biocontrol technologies gaining ground in sub-Saharan African countries, (iii) fine-tuning of mycotoxin biosynthesis at the level of transcription factors, and (iv) interference of mycotoxins with the normal physiological processes of liver and kidneys. We are pleased to recommend all these new publications to all professionals, university lecturers, students and other interested parties who want to get a comprehensive, authentic picture of the latest results and future trends related to *Aspergillus* mycotoxin research.

Today it seems unquestionable and irreversible that aflatoxigenic *Aspergillus* species are moving constantly north in Europe due to the climate change, which is an emerging threat to consumers. Advanced mycotoxin control and disinfection techniques are required to combat successfully such molds and their dangerous metabolites. Although effective techniques are available we need to explore their limitations to facilitate the development of future tools to address the growing world-wide aflatoxin risk adequately (Loi et al.).

The first large-scale survey of chronic aflatoxin M1 (AFM1) exposure of Hungarian consumers showed that AFM1 exposure of older age groups does not pose an emergent health risk, but younger age groups, especially toddlers, are exposed to a certain level of health risk. Further studies are awaited to provide a more detailed overview of the true aflatoxin health risk and exposure assessment of combined multi-mycotoxin contamination (Farkas et al.).

Lactic acid bacteria have always been considered among the promising microorganisms in inhibiting the growth and toxin production of mycotoxin-producing molds. Latest results reached by the probiotic *Lacticaseibacillus rhamnosus* C1 against *Penicillium roqueforti* further confirm this view in addition to the new observation that the antifungal effect of protocatechuic acid can be used in future technologies to effectively block the growth of any mycotoxin producer fungi (An et al.).

It is an attractive idea to develop and use atoxigenic *Aspaergillus* strains to control aflatoxigenic molds in Europe as well although there are still concerns about applying such agents in our continent (Loi et al.). Imporatntly, highly effective Aflasafe^®^ products have been developed, authorized or approved in many sub-Saharan African countries. Most recently, the distribution of aflatoxin contamination was mapped and the causal agents were identified in Burundi. Furthermore, some atoxigenic genotypes of *Aspergillus flavus* were detected across the country and four non-aflatoxigenic strains were selected as ingredients of the new biocontrol product Aflasafe BU01 (Nsabiyumva et al.). In addition to country-specific atoxigenic genotypes, registered ingredients of the Kenyan biocontrol product Aflasafe KE01 also frequently appear in maize and groundnut produced in Burundi, carrying the possibility that Aflasafe KE01 could be used across East Africa (Nsabiyumva et al.). In Nigeria, the biocontrol agent Aflasafe produced by the private sector has been shown to be effective in meeting the aflatoxin tolerance threshold in maize. These findings were presented and discussed at a National Workshop attended by all parties interested in ensuring strict and effective control of aflatoxin in the Nigerian food chain (Ola et al.).

A deeper understanding of the genetics of mycotoxin production is of paramount importance e.g., when novel, RNA interference-based, plant host induced control technologies are developed (Loi et al.). In the filamentous fungus model organism *Aspergillus nidulans*, interactions between the bZIP-type transcription factors AtfA and AtfB are likely when genes involved in stress tolerance, sexual and asexual developments as well as secondary metabolite (e.g., the aflatoxin-precursor sterigmatocystin) production are co-regulated by them. It is noteworthy that no sterigmatocystin production was observed in agar plate cultures of the *A. nidulans* Δ*atfA* gene deletion mutant meanwhile the Δ*atfB* mutant and the Δ*atfA* Δ*atfB* double mutant produced this mycotoxin nearly at wild-type level (Kocsis et al.).

The adverse effects of mycotoxins can be observed in several organs of both humans and domestic animals but liver and kidneys are particularly sensitive. Hepatic enzymes are key players in the metabolism of these toxic compounds, which is divided into Phases I (oxidation, hydrolysis, and reduction) and II (conjugation). In humans, phase I metabolism is mainly performed by cytochrome P450 (CYP450) enzymes, among which CYP1A2 and CYP3A4/3A5 enzymes contribute to the metabolism of AFB1 at 49 and 45%, respectively, as determined in pooled human live microsomes. The *in vitro* pharmacokinetic characterization of CYP1A2 and CYP3A4 may help to understand the interindividual variabilities observed in the activity of liver CYP450 enzymes (Lootens et al.).

Although kidneys are also remarkably vulnerable to the adverse effects of mycotoxins, farm animals are not generally screened yet for mycotoxin induced nephropathy. We suggest that changes in urinary biomarkers extensively used in environmental toxicology including β_2_-microglobulin (β_2_-MG), *N*-acetyl-β-D-glucosaminidase (NAG), kidney injury molecule 1 (KIM-1) and neutrophil gelatinase-associated lipocalin (NGAL) should also be monitored in mycotoxin-exposed livestock as in pigs, which are particularly susceptible to *Aspergillus*-derived mycotoxins (Ráduly et al.).

## Author contributions

All authors served as co-editors to the Research Topic and also contributed to, critically read, discussed, and approved this editorial.
